# The Age at Onset of LRRK2 p.Gly2019Ser Parkinson's Disease Across Ancestries and Countries of Origin

**DOI:** 10.1002/ana.78181

**Published:** 2026-02-24

**Authors:** Theresa Lüth, Björn‐Hergen Laabs, Sebastian Sendel, Inke R. König, Amke Caliebe, Alastair J. Noyce, Laurel A. Screven, Soraya Bardien, Matthew Farrer, Faycel Hentati, Rim Amouri, Christine Klein, Samia Ben Sassi, Joanne Trinh, Global Parkinson's Genetics Program (GP2)

**Affiliations:** ^1^ Institute of Neurogenetics University of Lübeck Lübeck Germany; ^2^ Institute of Medical Biometry and Statistics, University of Lübeck Lübeck Germany; ^3^ Department of Medical Statistics University Medical Center Göttingen Göttingen Germany; ^4^ Institute of Medical Informatics and Statistics, Kiel University and University Hospital Schleswig‐Holstein Kiel Germany; ^5^ Statistical Genetics and Clinical Studies, Medical Faculty, Health and Medical University Erfurt Erfurt Germany; ^6^ Centre for Preventive Neurology, Wolfson Institute of Population Health, Queen Mary University of London London UK; ^7^ National Institute on Aging Bethesda MD; ^8^ Division of Molecular Biology and Human Genetics Faculty of Medicine and Health Sciences, Stellenbosch University and South African Medical Research Council/Stellenbosch University Genomics of Brain Disorders Research Unit Cape Town South Africa; ^9^ Department of Neurology University of Florida Gainesville FL; ^10^ Service de Neurologie, Institut National de Neurologie La Rabta Tunis Tunisia; ^11^ National Institute Mongi Ben Hamida of Neurology Tunis Tunisia; ^12^ Faculty of Medicine of Tunis Tunis Tunisia; ^13^ Neurology's Department Mongi Ben Hmida National Institute of Neurology Tunis Tunisia

## Abstract

**Objectives:**

The objective of this study was to elucidate differences in the cumulative incidence of Leucine‐rich repeat kinase 2 (LRRK2) p.Gly2019Ser*‐*related Parkinson's disease (PD; *LRRK2*‐PD) between ancestries and countries.

**Methods:**

We included 922 unrelated p.Gly2019Ser variant carriers (affected = 762 and unaffected = 160) from the Global Parkinson's Genetics Program (GP2) in addition to cohorts recruited from the Israeli Ashkenazi Jewish and Tunisian Arab‐Berber population. Cox proportional hazard models were applied to examine differences in cumulative incidence across ancestry groups and countries. All analyses were adjusted for biological sex and were exploratory.

**Results:**

The median age at onset (AAO) of *LRRK2*‐PD was 5 years younger in the North African (hazard ratio [HR] = 1.48, 95% confidence interval [CI] = 1.18–1.86, *p* = 7.0 × 10^−4^) compared with the European ancestry group. In contrast, the median AAO was 5 years older in the Ashkenazi Jewish (HR = 0.61, 95% CI = 0.50–0.75, *p* = 4.0 × 10^−6^) compared with the European ancestry group. Additionally, patients from Israel (HR = 1.59, 95% CI = 1.30–1.39, *p* = 4.0 × 10^−6^) and Tunisia (HR = 2.57, 95% CI: 2.16–3.06, *P* < 2.0 × 10^−16^) had a median 5‐year and 10‐year younger AAO compared with patients from the United States, respectively. Last, when focusing only on individuals with an Ashkenazi Jewish background, patients from Israel still had a younger AAO than those from the United States (HR = 1.82, 95% CI = 1.48–2.24, *p* = 1.5 × 10^−8^). Analogously, assessing only patients from the United States, the Ashkenazi Jewish ancestry group still had an older AAO than the European ancestry group (HR = 0.51, 95% CI = 0.39–0.67, *p* = 1.3 × 10^−6^).

**Interpretation:**

Our results provide evidence that a person's genetic ancestry and country of origin are associated with the AAO of *LRRK2*‐PD. This highlights the potential impact of both genetic and environmental factors on *LRRK2*‐PD AAO. ANN NEUROL 2026;99:1394–1404

Parkinson's disease (PD) is the fastest‐growing neurodegenerative disorder, currently affecting close to 12 million patients worldwide.^1^ The etiology of PD is complex and multifactorial, and it is not yet completely understood. Environmental exposures, lifestyle, and genetics shape disease susceptibility.

Approximately 15% of patients with PD have an underlying monogenic (ie, patients in which PD is caused by a single pathogenic variant) or carry a strong *GBA1* risk variant.^2^ Among these, the Leucine‐rich repeat kinase 2 (LRRK2) p.Gly2019Ser variant is the most common cause for autosomal dominant forms of PD. It is estimated that globally, 1 to 2% of all patients with PD carry the LRRK2 p.Gly2019Ser variant.^2^, ^3^, ^4^ However, the frequency of the variant varies significantly, depending on the ancestral population. In Tunisian Arab Berbers, LRRK2 p.Gly2019Ser accounts for approximately 30 to 40%^5^, ^6^, ^7^ of the PD cases, followed by Israeli Ashkenazi Jews at approximately 18% of all PD cases.^8^, ^9^, ^10^

Although all p.Gly2019Ser variant carriers have the same genetic cause, they are not phenotypically homogeneous, which is evident in the varied age at onset (AAO) and disease severity across patients. In fact, not all persons who carry the p.Gly2019Ser variant will develop PD. Thus, reduced penetrance has been observed in many individuals carrying LRRK2 p.Gly2019Ser. Among unrelated mutation carriers, the risk of developing PD increases with age and has been initially estimated to be 28% at age 59 years, 51% at 69 years, and 74% at 79 years^11^ or even greater than 80% at 70 years.^6^ In contrast, analyses in first or second‐degree relatives of affected carriers using kin‐cohort analyses presented lower estimates of the LRRK2 p.Gly2019Ser penetrance at approximately 10 to 33% at 80 years.^12^, ^13^ Thus, different studies assessed the penetrance of LRRK2 p.Gly2019Ser, with varying results depending on cohort compositions (eg, families or unrelated mutation carriers) and statistical analysis approaches.^14^ In addition, there is evidence for ancestral population‐specific effects in LRRK2 p.Gly2019Ser‐related PD (*LRRK2*‐PD), as the penetrance is higher in the Arab‐Berber population compared with US Ashkenazi Jews and Europeans.^5^, ^13^ Although a lower disease risk was also reported for p.Gly2019Ser variant carriers from North Africa.^15^ Concerning disease onset, no difference has been shown in AAO of patients with *LRRK2*‐PD from the United States or Tunisia; however, with a limited sample size.^4^ In another study, Norwegian patients with *LRRK2*‐PD had an older AAO compared with patients from the Tunisian Arab‐Berber and Israeli Ashkenazi Jewish populations.^16^

A thorough assessment of the relationship between ancestry or the country of origin and *LRRK2*‐PD onset has not been performed. Thus, we herein utilize data from the Global Parkinson's Genetics Program (GP2)^17^ to assess the cumulative incidence in 922 unrelated LRRK2 p.Gly2019Ser variant carriers from different ancestries and countries. The GP2 cohort includes patients from all over the world, with a particular focus on under‐represented populations. Utilizing resources like the GP2 dataset can help to overcome biases arising from limited statistical power and lack of non‐European ancestry participants.

## Methods

### Study Cohort

We included 581 LRRK2 p.Gly2019Ser variant carriers from GP2, utilizing the genetic and clinical data of release 7 (https://gp2.org/).^17^ Additionally, we included LRRK2 p.Gly2019Ser variant carriers recruited from the Israeli Ashkenazi Jewish (N = 127), as previously described,^16^ and the Tunisian Arab Berber (N = 214) populations.

Ethical approval for the full study was obtained from the University of Lübeck. The details of the institutional review board (IRB)/oversight body that provided approval or exemption for the research are as follows: (1) data used in the preparation of this article were obtained from the GP2 (https://gp2.org). Specifically, we used tier 2 data from GP2 release 7 (https://doi.org/10.5281/zenodo.10962119). Tier 1 data can be accessed by completing a form on the Accelerating Medicines Partnership in Parkinson's Disease (AMP‐PD) website (https://amp-pd.org/register-for-amp-pd). Tier 2 data access requires approval and a Data Use Agreement signed by your institution. (2) Data from a cohort recruited from the Tunisian Arab Berber, and the study was approved by the local ethics committee of the Mongi Ben Hmida National Institute of Neurology. (3) Data from a published cohort recruited from the Israel Ashkenazi Jewish population were included, as described before.^16^

The GP2 genotyping data, stored in PLINK format,^18^ was utilized to extract the LRRK2 p.Gly2019Ser genotype (ie, chr12:40340400 [hg38], rs34637584). The genotype data included in GP2 is obtained from the NeuroBooster array^19^ and was processed for quality control to encompass call rate pruning, evaluations for discordant sex, detect duplicates or related individuals (kinship, defaults = 0.0884/0.354 related/duplicated), and heterozygosity rates. Genotype imputation was performed through the TOPMed server.^20^ All individuals were stratified into different ancestry groups by using the diverse reference panel based on prediction from the principal component analysis by GenoTools,^21^ resulting in a classification of the individuals by GP2 into the following ancestry groups: African (AFR), African admixed (AAC), Ashkenazi Jewish (AJ), Latino and Indigenous people of the Americas (AMR), East Asian (EAS), European (EUR), South Asian (SAS), Central Asian (CAS), Middle Eastern (MDE), Finnish (FIN), and Complex Admixture (CAH) (Supplementary Fig S1). However, for the purposes of our study, we re‐classified individuals from Tunisia (TUN) who were assigned to the MDE ancestry group to the North African ancestry group (NA). Subsequently, the genotyping data were merged with the clinical metadata to obtain information on affection status, age (defined as age at sample collection), biological sex, and country of origin (original region/country of the sample; missing values will be replaced by the region of the central institution). Reported AAO (defined as the age at first PD symptom) was used. For 83 participants that lacked AAO information, the age at PD diagnosis was used instead (Supplementary Table S1). We only included ancestry groups with at least 10 unrelated LRRK2 p.Gly2019Ser variant carriers with complete clinical data (ie, AJ, AMR, EUR, MDE, and NA).

In order to explore whether the observed difference in cumulative incidence across ancestries was specific to the group of patients with *LRRK2*‐PD, we additionally included patients with PD who did not carry the LRRK2 p.Gly2019Ser variant (non‐*LRRK2*‐PD) from the GP2 dataset. In total, we included 10,892 patients with non‐*LRRK2*‐PD that were from the AJ (N = 1,029) and EUR (N = 9,863) ancestry groups. In line with the LRRK2 variant carriers from GP2, the non‐*LRRK2*‐PD patients were also genotyped using the NeuroBooster array, providing information about the p.Gly2019Ser status.

To minimize bias from related individuals, only unrelated LRRK2 p.Gly2019Ser variant carriers were included in the analyses. For GP2 participants, relatedness and duplicate information were provided by GP2. In brief, the standard GenoTools pipeline estimates pairwise kinship coefficients based on genotyping data (default thresholds = 0.0884 for related and 0.354 for duplicated samples).^21^ Identified related or duplicate samples were excluded. For the Tunisian Arab Berber and AJ cohorts, familial relationships were provided in the clinical metadata and used to exclude related individuals. In total, up to third‐degree relatives were removed from the study cohort.

### Statistical Analysis

All analyses and data visualization were conducted with the statistical software R (R version 4.3.1).^22^ We utilized the Cox proportional hazards model to assess the differences in cumulative incidence across different ancestries and countries and included affected and unaffected variant carriers. The proportional hazards assumption was evaluated graphically using log(−log(survival probability)) versus log(time) plots. Visual inspection indicated no substantial deviations from parallelism, supporting the validity of the proportional hazards assumption (Supplementary Fig S2). In the model, age (for unaffected variant carriers, censored) or AAO (for affected variant carriers), together with the affected/unaffected status, was the dependent variable, and ancestry and/or country of origin were the independent variables. Thus, event time was defined to be AAO with unaffected carriers being censored at their current age; and events were defined as affection with PD. The model was also adjusted for sex, given the unbalanced men‐to‐women ratios in some ancestry groups and the potential younger AAO of female patients with *LRRK2*‐PD.^5^ The models were calculated with the R package *survival* (version 3.5‐7)^23^ and displayed as Forrest plots with the R package *forestmodel* (version 0.6.2, https://CRAN.R-project.org/package=forestmodel). Last, age‐associated cumulative incidences across ancestries and countries were estimated using Cox proportional hazards models adjusted for sex. Model‐based survival curves derived from these fitted models were visualized to illustrate sex‐adjusted cumulative incidence patterns. The plots were visualized with the R package *survminer* (version 0.4.9, https://CRAN.R-project.org/package=survminer). All analyses were exploratory, and *p* values cannot be interpreted for significance.

## Results

In order to investigate the cumulative incidence of *LRRK2*‐PD across ancestries and countries of origin, we assessed 922 LRRK2 p.Gly2019Ser variant carriers (762 affected and 160 unaffected). The variant carriers belonged to 5 different ancestries: EUR, AJ, AMR, MDE, and NA (see the Table 1). The cumulative incidence across the entire cohort showed a median AAO of 60 years (Supplementary Fig S3). The cumulative incidence of *LRRK2*‐related PD was 25% (95% CI = 22–27%) by age 50 years, 50% (95% CI = 47–54%) by age 60 years, 81% (95% CI = 78–84%) by age 70 years, and 95% (95% CI = 93–97%) by age 80 years in the study cohort.

**TABLE 1:** Demographics of LRRK2 p.Gly2019Ser Variant Carriers

	N	N of Men (%)	Countries of Origin Distribution (N)	N of Patients With PD and Mean AAO (SD)	N of Unaffected Carriers and Mean AAE (SD)
AJ	534	273 (51.1)	USA (N = 354), ISR (N = 157), IND (N = 15), ZAF (N = 4), GBR (N = 3), NZL (N = 1)	395 59.81 yr (11.32)	139 61.90 yr (11.35)
NA	223	116 (52.0)	TUN (223)	223 52.56 years (12.38)	‐
EUR	132	66 (50.0)	USA (N = 86), ESP (N = 22), FRA (N = 7), NZL (N = 6), GBR (N = 4), DEU (N = 2), AUS (N = 1), CAN (N = 1), CHL (N = 1), IND (N = 1), ZAF (N = 1)	115 56.95 yr (11.32)	17 49.84 yr (13.40)
MDE	19	16 (84.2)	FRA (N = 12), ESP (N = 3), USA (N = 2), CAN (N = 1), DEU (N = 1)	17 45.71 yr (9.56)	2 51.00 yr (28.28)
AMR	14	6 (42.9)	USA (N = 8), CAN (N = 2), CHL (N = 2), AZE (N = 1), FRA (N = 1)	12 57.25 yr (14.42)	2 58.76 yr (13.07)
Total	922	477 (51.7)	‐	762 56.90 yr (12.16)	160 60.44 yr (12.29)

AAE = age at examination; AAO = age at onset; AJ = Ashkenazi Jewish ancestry; AMR = Latino and Indigenous Americas populations; AUS = Australia; AZE = Azerbaijan; CAN = Canada; CHL = Chile; DEU = Germany; ESP = Spain; EUR = General European ancestry; FRA = France; GBR = Great Britain United Kingdom; IND = India; ISR = Israel; MDE = Middle Eastern ancestry; N = number of individuals; NA = North African ancestry; NZL = New Zealand; PD = Parkinson's disease; TUN = Tunisia; USA = United States of America; ZAF = South Africa.

### *LRRK2* ‐PD Cumulative Incidence Across Different Ancestries

We observed differences between the AAO of patients with *LRRK2*‐PD across the ancestries (see the Table 1). The mean (SD) AAO was the oldest in the AJ ancestry group (59.81 [±11.32] years) and youngest in the MDE group (45.71 [±9.56] years). To statistically assess the relationship between ancestries and AAO of *LRRK2*‐PD, we performed a Cox proportional hazards analysis (Supplementary Fig S4). We used EUR ancestry (N = 132) as a reference and observed an older AAO in the AJ ancestry group (N = 534), a younger AAO in the NA (N = 223) and MDE (N = 19) ancestry groups, and no difference in AAO in the AMR ancestry group (N = 14). To avoid potential biases arising from too small sample sizes, we focus our analysis on the 3 largest ancestry groups in our study cohort (ie, EUR, NA, and AJ). Because there was no specific predefined a priori hypothesis, all reported *p* values are solely exploratory.

**FIGURE 1:**
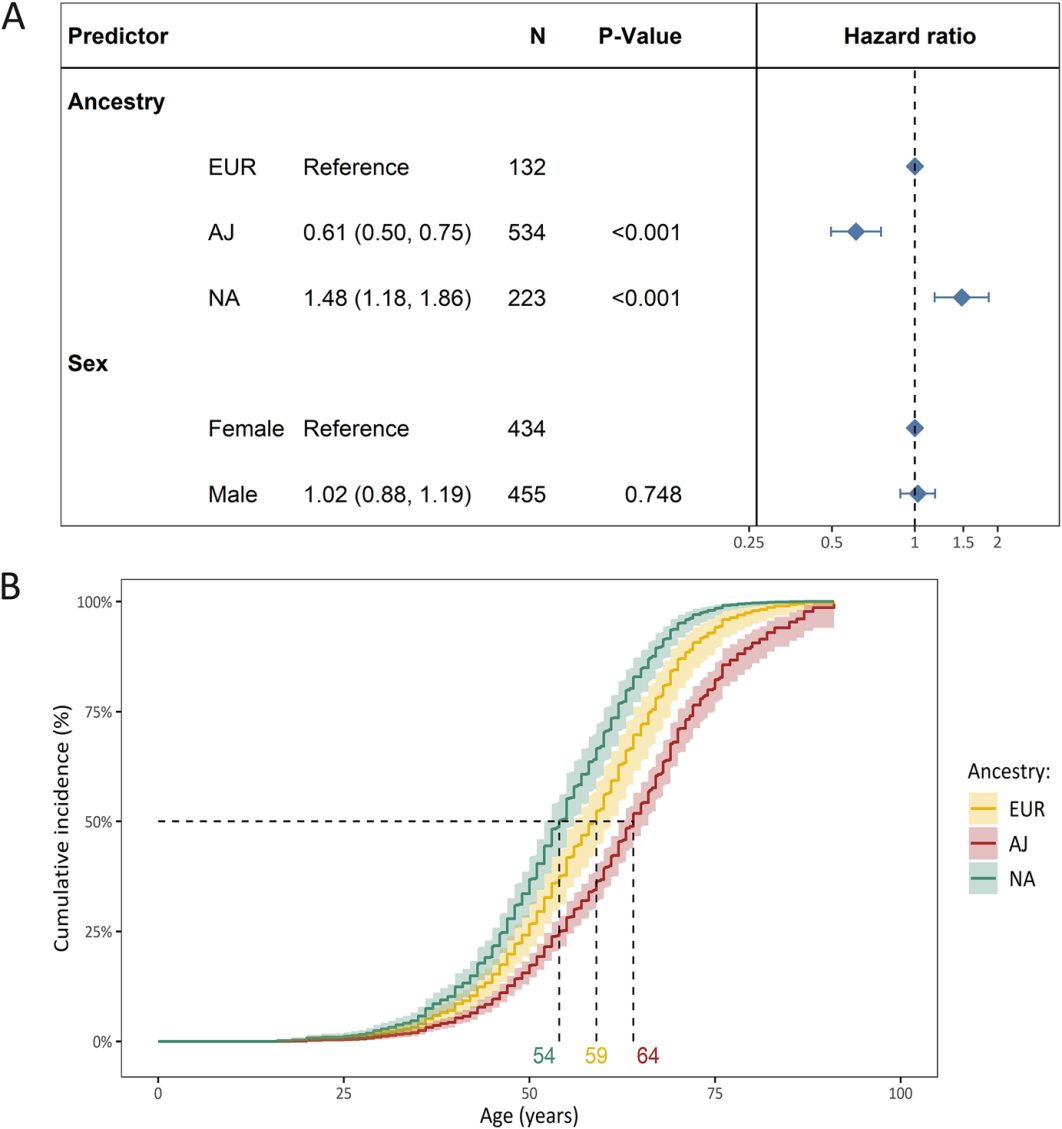
The difference in cumulative incidence of LRRK2 p.Gly2019Ser variant carriers from different genetic ancestries. (A) The forest plot indicates the difference in cumulative incidence of different ancestries where the hazard ratios, confidence intervals, and *p* values were derived from a Cox proportional hazards model, adjusted for sex. The reference category of the assessed ancestries was set to European ancestry (EUR). Affected and unaffected LRRK2 p.Gly2019Ser variant carriers were included in the model and the outcome was age at onset or age at examination with right censoring of the affection status. (B) Model‐based survival curves derived from the sex‐adjusted Cox proportional hazards model are shown. These curves represent sex‐adjusted cumulative incidence estimates across ancestries and the 95% confidence interval. Age = age at examination or age at onset; AJ = Ashkenazi Jewish ancestry; EUR = General European ancestry; N = number of individuals; NA = North African ancestry; PD = Parkinson's disease. [Color figure can be viewed at www.annalsofneurology.org]

In comparison to the EUR ancestry group, the median AAO was older in the AJ ancestry group (hazard ratio [HR] = 0.61, 95% confidence interval [CI] = 0.50–0.75, *p* = 4.0 × 10^−6^) and younger in the NA ancestry group (HR = 1.48, 95% CI = 1.18–1.86, *p* = 7.0 × 10^−4^; Fig 1A). The sex‐adjusted median age of survival without PD was 10 years younger in the NA ancestry group (median age = 54 years, 95% CI = 52.9–56) compared with the AJ ancestry group (median age = 64 years, 95% CI = 52.4–66) and 5 years younger compared with the EUR ancestry group (median age = 59 years, 95% CI = 56–61; Fig 1B).

### *LRRK2* ‐PD Cumulative Incidence Across Different Countries of Origin

Next, we investigated the relationship between the country of origin and the cumulative incidence of *LRRK2*‐PD. There were 6 countries with at least 10 unrelated LRRK2 p.Gly2019Ser variant carriers: the United States (N = 457), Tunisia (TUN; N = 223), Israel (ISR; N = 157), Spain (ESP; N = 25), France (FRA; N = 20), and India (IND; N = 16). We observed differences between the median AAO of patients with *LRRK2*‐PD across the countries of origin. To statistically assess these differences, we also performed a Cox proportional hazards analysis (Supplementary Fig S5). We used the United States as a reference and observed a younger AAO in participants from ESP, FRA, ISR, and TUN. There was no difference compared with participants from IND. To avoid potential biases arising from too small sample sizes again, we focused our analysis on the 3 countries with the largest sample sizes in our study cohort (ie, the United States, TUN, and ISR).

**FIGURE 2:**
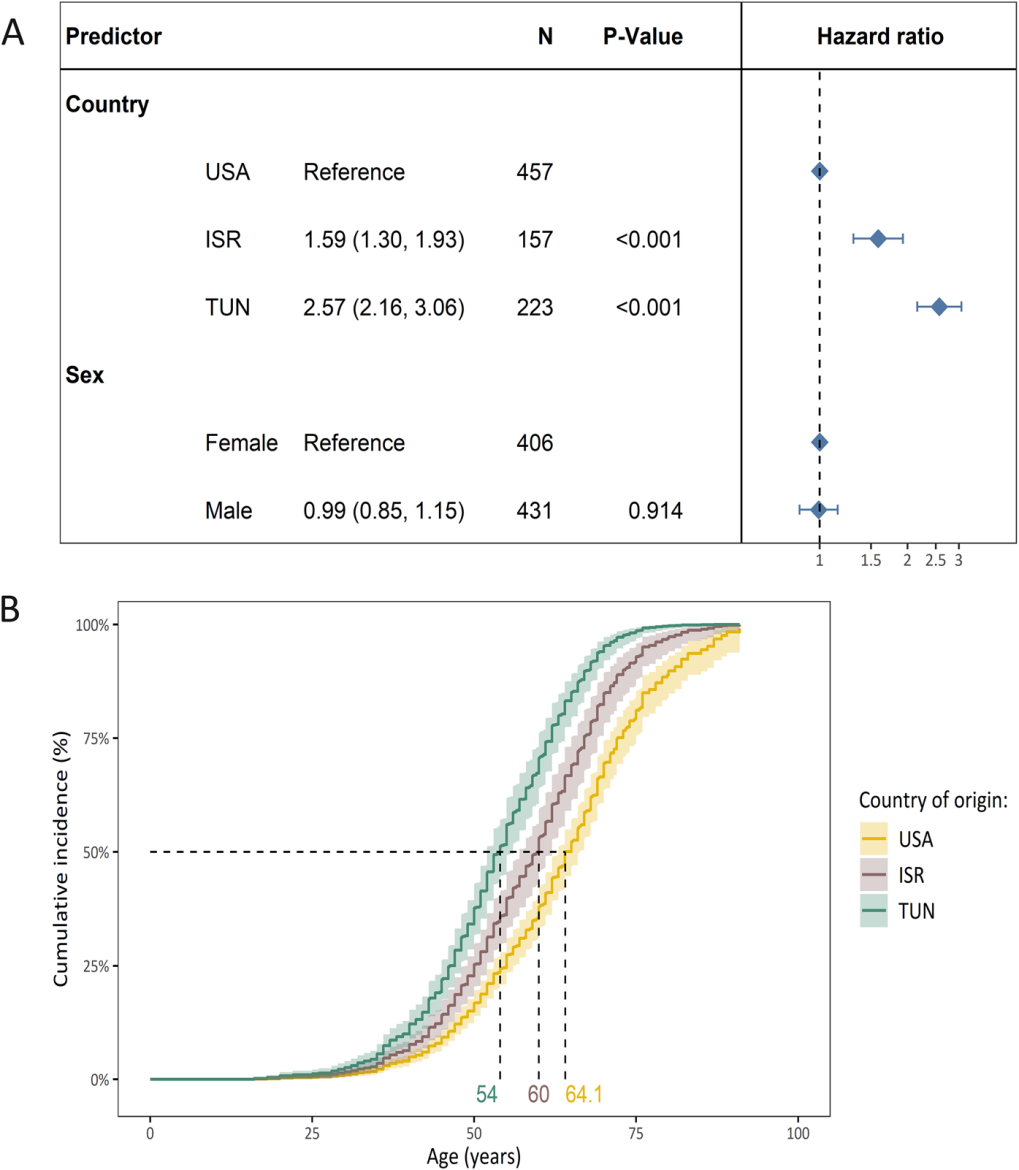
The difference in cumulative incidence of LRRK2 p.Gly2019Ser variant carriers from different countries of origin. (A) The forest plot indicates the difference in cumulative incidence from different countries where the hazard ratios and *p* values were derived from a Cox proportional hazards model, adjusted for sex. The reference category of the assessed countries was set to the United States. Affected and unaffected LRRK2 p.Gly2019Ser variant carriers were included in the model and the outcome was age at onset or age at examination with right censoring of the affection status. (B) Model‐based survival curves derived from the sex‐adjusted Cox proportional hazards model are shown. These curves represent sex‐adjusted cumulative incidence estimates across countries and the 95% confidence interval. Age = age at examination or age at onset; ISR = Israel; N = number of individuals; PD = Parkinson's disease; TUN = Tunisia; USA = United States of America. [Color figure can be viewed at www.annalsofneurology.org]

In comparison to the persons from the United States, the median AAO was younger among persons from ISR (HR = 1.59, 95% CI = 1.30–1.39, *p* = 4.0 × 10^−6^) and also younger among persons from TUN (HR = 2.57, 95% CI = 2.16–3.06, *p* < 2.0 × 10^−16^; Fig 2A). The sex‐adjusted median age of survival without PD was 10 years younger in variant carriers from TUN (median age = 54 years, 95% CI = 52–56) compared with variant carriers from the United States (median age = 64.1 years, 95% CI = 63–66.3) and 6 years younger compared with individuals from ISR (median age = 60 years, 95% CI = 57–62; Fig 2B).

### Ancestries and Country of Origin Drive the Difference in Cumulative Incidence

Next, we investigated if the difference in cumulative incidence of *LRRK2*‐PD is driven by ancestry or country of origin. Thus, we focused on the ancestry group and country with the most included variant carriers, which were AJ and the United States, respectively.

**FIGURE 3:**
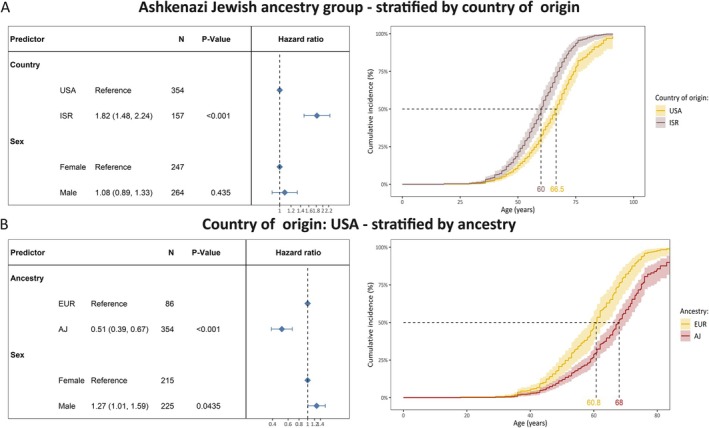
The difference in cumulative incidence of LRRK2 p.Gly2019Ser variant carriers from different countries of origin and ancestries. (A) The forest plot indicates the difference in cumulative incidence from different countries where the hazard ratios and *p* values were derived from a Cox proportional hazards model, adjusted for sex. The reference category of the assessed countries was set to the United States. Affected and unaffected LRRK2 p.Gly2019Ser variant carriers were included in the model and the outcome was age at onset or age at examination with right censoring of the affection status. Model‐based survival curves derived from the sex‐adjusted Cox proportional hazards model are shown. These curves represent sex‐adjusted cumulative incidence and the 95% confidence interval. All included mutation carriers were of Ashkenazi Jewish ancestry. (B) The forest plot indicates the difference in cumulative incidence from different ancestries where the hazard ratios and *p* values were derived from a Cox proportional hazards model, adjusted for sex. The reference category of the assessed countries was set to EUR. Affected and unaffected LRRK2 p.Gly2019Ser variant carriers were included in the model and the outcome was age at onset or age at examination with right censoring of the affection status. Model‐based survival curves derived from the sex‐adjusted Cox proportional hazards model are shown. These curves represent sex‐adjusted cumulative incidence and the 95% confidence interval. All included mutation carriers originated from the United States. Age = age at examination or age at onset; AJ = Ashkenazi Jewish ancestry; EUR = General European ancestry; ISR = Israel; N = number of individuals; PD = Parkinson's disease; USA = United States of America. [Color figure can be viewed at www.annalsofneurology.org]

To test if the country of origin is associated with AAO beyond the genetic ancestry, we performed the Cox proportional hazards analysis only in the AJ ancestry group. Within the AJ ancestry group, there were 354 individuals from the United States and 157 individuals from ISR. Notably, although all persons belonged to the same ancestry group, the median AAO was younger in patients from ISR (HR = 1.82, 95% CI = 1.48–2.24, *p* = 1.5 × 10^−8^). The median AAO was 7 years younger in variant carriers from ISR (median age = 60 years, 95% CI = 58.7–62.1) compared with variant carriers from the United States (median age = 66.5 years, 95% CI = 65–69; Fig 3A).

On the other hand, to test if ancestry is associated with AAO beyond the country of origin, we performed the Cox proportional hazards analysis only in persons from the United States. Within the group of individuals from the United States, there were 86 individuals with EUR ancestry and 354 with AJ ancestry. Although all persons were from the same country, the median AAO was older in patients from the AJ ancestry group (HR = 0.51, 95% CI = 0.39–0.67, *p* = 1.3 × 10^−6^). The median AAO was approximately 7 years older in variant carriers from the AJ ancestry group (median age = 68 years, 95% CI = 66–70.2) compared with variant carriers from the EUR ancestry group (median age = 60.8 years, 95% CI = 59–64; Fig 3B).

Furthermore, we applied a Cox proportional hazards model with both country and ancestry as independent variables, including variant carriers from the EUR and AJ ancestry group as well as from ISR and the United States. Still, the countries of origin and ancestries remained associated with cumulative incidence (Supplementary Fig S6). In fact, individuals from the AJ ancestry group still had an older median AAO compared with individuals from the EUR group (HR = 0.52, 95% CI = 0.40–0.68, *p* = 2.3 × 10^−6^), and individuals from ISR still had a younger median AAO compared with individuals from the United States (HR = 1.81, 95% CI = 1.47–2.23, *p* = 1.8 × 10^−8^).

### Cumulative Incidence Across Different Ancestries of Patients With PD That Do Not Carry the LRRK2 p.Gly2019Ser Variant

Last, we explored patients with PD who did not carry the LRRK2 p.Gly2019Ser variant (non‐*LRRK2*‐PD). We focused our analysis on the ancestry groups relevant to this study. In total, we included 10,892 patients with non‐*LRRK2*‐PD from the AJ (*N* = 1,029) and EUR (*N* = 9,863) ancestry groups, where most of the patients were from the United States. Compared with patients with *LRRK2*‐PD, the median AAO of patients with non‐*LRRK2*‐PD was approximately 2 years older in both the EUR (HR = 0.82, 95% CI = 0.68–0.98, *p* = 0.033) and AJ (HR = 0.80, 95% CI = 0.72–0.90, *P* = 2.7 × 10^−4^) ancestry groups.

To assess whether the observed difference in cumulative incidence across ancestries was specific to the group of patients with *LRRK2*‐PD, we analyzed patients without the p.Gly2019Ser variant. Analogous to the patients with *LRRK2*‐PD, patients who did not carry the p.Gly2019Ser variant and belonged to the EUR ancestry group had a younger median AAO than those from the AJ ancestry group, albeit with a smaller effect size (HR = 0.78, 95% CI = 0.73–0.83, *p* = 1.4 × 10^−14^). There was a 3‐year difference in the median AAO of patients with non‐*LRRK2*‐PD from the EUR (median AAO = 60 years) and AJ (median AAO = 63 years) ancestry groups (Supplementary Fig S7). The sample size of patients with non‐*LRRK2*‐PD from the NA ancestry group, Tunisia, or Israel was too small to allow for other ancestral or country‐related comparisons.

## Discussion

In this study, we explored the relationship among genetic ancestry, country of origin, and the cumulative incidence of *LRRK2*‐PD. Utilizing one of the largest and most ancestral diverse study cohorts of LRRK2 p.Gly2019Ser variant carriers, we found evidence that both country of origin and genetic ancestry are associated with the cumulative incidence. Our key findings are: (1) individuals of North African genetic ancestry had a median of 5 years younger AAO than those from the EUR group, (2) conversely, individuals of AJ ancestry had a 5‐year older median AAO compared with the EUR group, and (3) at the country level, patients from TUN and ISR had a 10‐year and 5‐year younger median AAO, respectively, compared with patients from the United States.

The median AAO was the oldest in the AJ ancestry group, followed by the EUR ancestry group and the youngest in the NA ancestry group. Thus, although all patients presumably had the same underlying monogenic cause of their disease, the ancestries’ different genetic backgrounds might affect the AAO. It is known that genetic modifiers are associated with disease risk and AAO in monogenic forms of PD. For *LRRK2*‐PD in particular, although not yet replicated, *CORO1C* and *DNM3* have been nominated as genetic modifiers.^24^, ^25^ Additionally, it has been demonstrated recently that a higher PD polygenic risk score is associated with a higher penetrance among LRRK2 p.Gly2019Ser variant carriers.^26^, ^27^ Associations between ancestry and disease onset or disease risk have been observed in neurodegenerative diseases like Alzheimer's disease and amyotrophic lateral sclerosis.^28^, ^29^

We have previously assessed the implications of ancestry on the *LRRK2*‐PD AAO and showed that Norwegians had a significantly later AAO compared with Tunisian Arab Berber and Israeli Ashkenazi Jewish populations.^16^ Our data in this study could further highlight the large potential impact of genetic ancestry on the *LRRK2*‐PD AAO. However, in our dataset, the EUR ancestry group had an approximately 5 years younger median AAO compared with the AJ group. This discrepancy can result from the composition of individuals in the AJ ancestry group, as they were from the United States and Israel, whereas the previous study was limited to individuals from Israel. Additionally, individuals with EUR ancestry in this study here did not originate from Norway but are from various countries (eg, the United States, France, or Spain).

Therefore, we next explored the relationship between countries and cumulative incidence. In contrast to ancestry, the country of origin is not derived from a person's genetic background. The country of origin might reflect similar environmental exposures or lifestyle preferences of persons living in the same geographic region. Still, it is important to note that the individual exposures can vary strongly between individuals within one country. In our dataset, individuals from the United States had the oldest median AAO. Individuals from TUN had an approximately 10‐year younger median AAO, and individuals from ISR had approximately 5 years younger median AAO compared with individuals from the United States. Indeed, the importance of environmental and lifestyle factors for PD susceptibility and the association with *LRRK2*‐PD AAO is known.^30^, ^31^ Thus, it is reasonable that the country of origin is also associated with disease onset, although potential recruitment bias across the various countries might also impact this finding.

It is important to note that, in our study cohort, the ancestry group and country of origin are strongly correlated and subsequently not independent. To explore whether one or the other is driving the observed difference in cumulative incidences, we assessed the impact of the country within one ancestry group (ie, AJ) and the impact of the ancestry within individuals from the same country (ie, the United States; see Fig 3). Importantly, the country and the ancestry were still associated with AAO. Thus, both might impact the AAO of *LRRK2*‐PD. However, replicating this analysis within other countries and ancestries would be required to confirm this finding. Additionally, potential joint interaction effects between countries and genetic ancestry groups are possible, but given the strong correlation between country of origin and genetic ancestry group in our study, they cannot be investigated with this dataset.

Interestingly, analogous to the patients with *LRRK2*‐PD, patients who did not carry the p.Gly2019Ser variant and belonged to the EUR ancestry group had a younger median AAO than those from the AJ ancestry group. However, the difference in non‐*LRRK2*‐PD was less pronounced, with only a 3‐year difference in median AAO. This observation might indicate that the ancestry‐related difference in AAO was not limited to LRRK2 p.Gly2019Ser variant carriers, and thus, our findings might be of importance to different PD subtypes.

In this study, we did not observe that the biological sex was associated with *LRRK2*‐PD AAO (Figs 1, 2). Biological sex differences associated with *LRRK2*‐PD AAO have been controversial in the literature. Female patients presented with an approximately 5 years younger AAO in TUN,^5^ and in AJ, there was no such difference.^13^ The difference in AAO of male and female patients with *LRRK2*‐PD likely depends on the sampling ancestry group and country of origin, which should be explored further in future studies. For example, cultural and gender‐related preferences of lifestyle factors might impact the difference in AAO of TUN male and female patients with *LRRK2*‐PD. One of the strongest protective lifestyle factors modifying PD risk and AAO is tobacco use, and the prevalence of smoking is substantially higher in TUN men compared with women.^32^

Besides genetics, lifestyle, and environmental exposure, access to specialized health care and differences in socioeconomic factors are known to impact PD risk and AAO,^33^ which likely contributed to the ancestry and country‐specific AAO differences we observed. The main limitation of our study is that no information about environmental, lifestyle, socioeconomic factors, or access to specialized health care was available. Therefore, these factors could not be included in our statistical analysis, resulting in potential unaccounted biases. Thus, future studies should investigate the ancestry‐specific effects of environment and lifestyle on *LRRK2*‐PD AAO. Currently, lacking environmental and lifestyle data within GP2 restricts such analyses, but comprehensive efforts are underway to address these gaps.

The country of origin is denoted as the original region/country of the sample. This information may not always accurately reflect an individual's lifetime environmental exposure. Participants may have moved one or several times during their lives, resulting in differing durations of residence and, consequently, varying environmental influences. Moreover, in rare cases within the GP2 cohorts, the region of the recruiting institution will be used. Therefore, whereas the country‐based stratification provides a useful framework for comparing populations, the interpretation of environmental effects should be made with caution. Movement between countries of interest for this study (ie, ISR, TUN, and the United States) is diplomatically challenging and is unlikely to occur in a significant manner relevant to the presented analysis. Future studies integrating more detailed data on the environmental exposures, lifestyle, place of birth, migration history, and duration of residence will be crucial to disentangle environmental from genetic contributions to the observed population‐level differences. Additionally, there could be differences in patient assessments, recruitment, and reported AAO between the different clinical sites within GP2 that we cannot be accounted for. Furthermore, there was a large variability in the number of unaffected variant carriers among the ancestry groups. Survival analyses are also not adapted to case–control data and would benefit from prospective longitudinal studies. To exclude potential recruitment bias reflected in the unbalanced number of unaffected variant carriers and avoid biases from the interpretation of the statistical analysis, we repeated the Cox proportional hazards model and only included affected patients. Still, the associations among ancestry, country, and cumulative incidence remained unchanged (Supplementary Fig S8). Additionally, we performed a sensitivity analysis excluding the 83 patients with *LRRK2*‐PD for whom only the age at diagnosis (AAD) was available instead of the AAO. This analysis revealed the same differences across ancestry groups and countries (Supplementary Fig S9), indicating that substituting AAO with AAD in these patients did not introduce any bias. Another limitation is that, in contrast to the AJ, EUR, and NA ancestry groups, the sample size of the AMR and MDE ancestries was too small to include them in the statistical analysis meaningfully. To achieve a more comprehensive understanding of *LRRK2*‐PD, it would be valuable to enhance the representation of participants from these ancestries in research programs. Still, our study cohort presents the most extensive data set of LRRK2 p.Gly2019Ser variant carriers to explore the cumulative incidence across different ancestries and countries.

In conclusion, our study provides evidence that a person's genetic ancestry and country of origin might be associated with the AAO of LRRK2 p.Gly2019Ser‐related PD. To reflect the exploratory nature of our work, these findings should be interpreted with appropriate caution and viewed as a first indication of potential associations. Future studies should aim to elucidate the underlying genetic, environmental, and socioeconomic factors, as well as potential gene–environment interactions, that may contribute to the observed AAO differences.

## Author Contributions

T.L., C.K., S.B.S., and J.T. contributed to the conception and design of the manuscript; T.L., B.H.L., S.S., I.R.K., A.C., A.J.N., L.S., S.B., M.F., F.H., R.A., C.K., S.B.S., and J.T. contributed to the acquisition and/or interpretation of the data included in the manuscript; T.L. and J.T. contributed to drafting the text and preparing the figures.

## Potential Conflicts of Interest

The authors declare that they have no conflict of interest.

## Supporting information


**Supplementary Table S1.** Fraction of LRRK2‐PD patients with only age at diagnosis information available.
**Supplementary Figure S1.** Principal component analysis (PCA).
**Supplementary Figure S2.** Log–log plots to test the proportional hazards assumption.
**Supplementary Figure S3.** The cumulative incidence of LRRK2 p.Gly2019Ser variant carriers.
**Supplementary Figure S4.** The difference in cumulative incidence of LRRK2 p.Gly2019Ser variant carriers from different genetic ancestries.
**Supplementary Figure S5.** The difference in cumulative incidence of LRRK2 p.Gly2019Ser variant carriers from different countries of origin.
**Supplementary Figure S6.** The difference in cumulative incidence of LRRK2 p.Gly2019Ser variant carriers from different genetic ancestries and countries.
**Supplementary Figure S7.** The difference in cumulative incidence of PD patients that do not carry the LRRK2 p.Gly2019Ser from different genetic ancestries.
**Supplementary Figure S8.** The difference in cumulative incidence of LRRK2 p.Gly2019Ser variant carriers from different genetic ancestries and countries.
**Supplementary Figure S9.** The difference in cumulative incidence of LRRK2 p.Gly2019Ser variant carriers from different genetic ancestry groups and countries of origin.

## Data Availability

Data used in the preparation of this article were obtained from the Global Parkinson's Genetics Program (GP2; https://gp2.org). Specifically, we used tier 2 data from GP2 release 7 (https://doi.org/10.5281/zenodo.10962119). Tier 1 data can be accessed by completing a form on the Accelerating Medicines Partnership in Parkinson's Disease (AMP‐PD) website (https://amp-pd.org/register-for-amp-pd). Tier 2 data access requires approval and a Data Use Agreement signed by your institution. All code generated for this article, and the identifiers for all software programs and packages used, are available on GitHub [https://github.com/GP2code/AAO_LRRK2_pG2019S] and were given a persistent identifier via Zenodo [https://github.com/GP2code/AAO_LRRK2_pG2019S].
