# Supplementary material for: The Age at Onset of LRRK2 p.Gly2019Ser Parkinson's Disease Across Ancestries and Countries of Origin
Source: Ann Neurol. 2026 Feb 24;99(6):1394–404. doi: 10.1002/ana.78181 (PMC13206296; doi:10.1002/ana.78181)
Supplement: Supplementary file 1 — Supplementary Table S1. Fraction of LRRK2‐PD patients with only age at diagnosis information available. Supplementary Figure S1. Principal component analysis (PCA). Supplementary Figure S2. Log–log plots to test the proportional hazards assumption. Supplementary Figure S3. The cumulative incidence of LRRK2 p.Gly2019Ser variant carriers. Supplementary Figure S4. The difference in cumulative incidence of LRRK2 p.Gly2019Ser variant carriers from different genetic ancestries. Supplementary Figure S5. The difference in cumulative incidence of LRRK2 p.Gly2019Ser variant carriers from different countries of origin. Supplementary Figure S6. The difference in cumulative incidence of LRRK2 p.Gly2019Ser variant carriers from different genetic ancestries and countries. Supplementary Figure S7. The difference in cumulative incidence of PD patients that do not carry the LRRK2 p.Gly2019Ser from different genetic ancestries. Supplementary Figure S8. The difference in cumulative incidence of LRRK2 p.Gly2019Ser variant carriers from different genetic ancestries and countries. Supplementary Figure S9. The difference in cumulative incidence of LRRK2 p.Gly2019Ser variant carriers from different genetic ancestry groups and countries of origin. [file ANA-99-1394-s001.docx]

**Content of Supplement**

Supplementary Table 1. Fraction of LRRK2-PD patients with only age at diagnosis information available.

Supplementary Figure 1. Principal component analysis (PCA).

Supplementary Figure 2. Log-log plots to test the proportional hazards assumption.

Supplementary Figure 3. The cumulative incidence of LRRK2 p.Gly2019Ser variant carriers.

Supplementary Figure 4. The difference in cumulative incidence of LRRK2 p.Gly2019Ser variant carriers from different genetic ancestries.

Supplementary Figure 5. The difference in cumulative incidence of LRRK2 p.Gly2019Ser variant carriers from different countries of origin.

Supplementary Figure 6. The difference in cumulative incidence of LRRK2 p.Gly2019Ser variant carriers from different genetic ancestries and countries.

Supplementary Figure 7. The difference in cumulative incidence of PD patients that do not carry the LRRK2 p.Gly2019Ser from different genetic ancestries.

Supplementary Figure 8. The difference in cumulative incidence of LRRK2 p.Gly2019Ser variant carriers from different genetic ancestries and countries.

Supplementary Figure 9. The difference in cumulative incidence of LRRK2 p.Gly2019Ser variant carriers from different genetic ancestry groups and countries of origin

**Supplementary Table 1.** Fraction of *LRRK2*-PD patients with only age at diagnosis information available.

|  | ***N* of patients with PD** | ***N* of patients with PD & only AAD information available (%)** |
| --- | --- | --- |
| AJ | 395 | 73 (18.5%) |
| NA | 223 | --- |
| EUR | 115 | 7 (6.1%) |
| MDE | 17 | --- |
| AMR | 12 | 3 (25.0%) |
| **Total** | **762** | **83 (10.9%)** |

N=Number of individuals, AAD=Age at diagnosis, PD=Parkinson’s disease, AJ= Ashkenazi Jewish ancestry, AMR=Latino and Indigenous Americas populations, MDE=Middle Eastern ancestry, EUR=General European ancestry, NA=North African ancestry


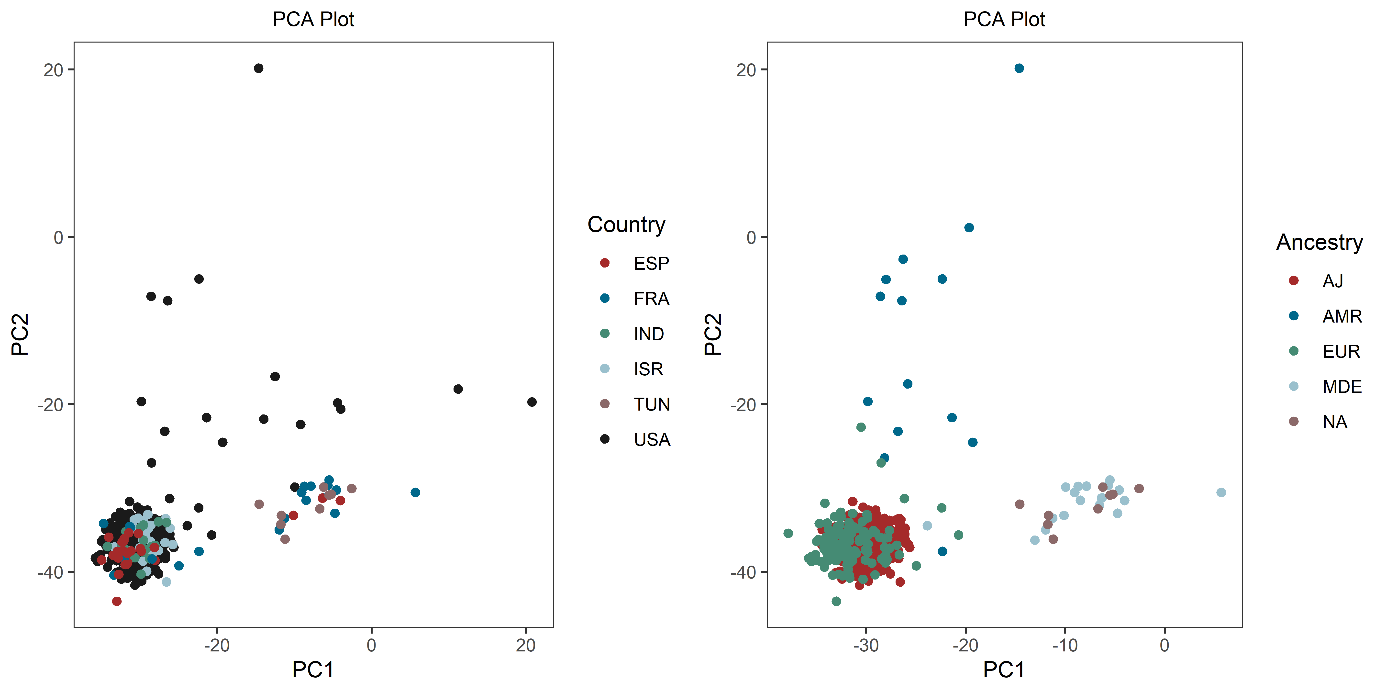


**Supplementary Figure 1. Principal component analysis (PCA).** The PCA plot displays the clustering of LRRK2 p.Gly2019Ser variant carriers of the GP2 dataset included in this study.

AJ=Ashkenazi Jewish ancestry, AMR=Latino and indigenous Americas populations, MDE=Middle Eastern ancestry, EUR=General European ancestry, NA=North African ancestry, ESP=Spain, FRA=France, IND=India, ISR=Israel, USA=United States of America, TUN=Tunisia.


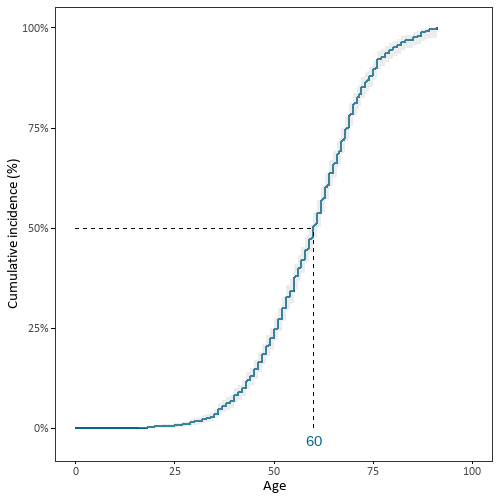

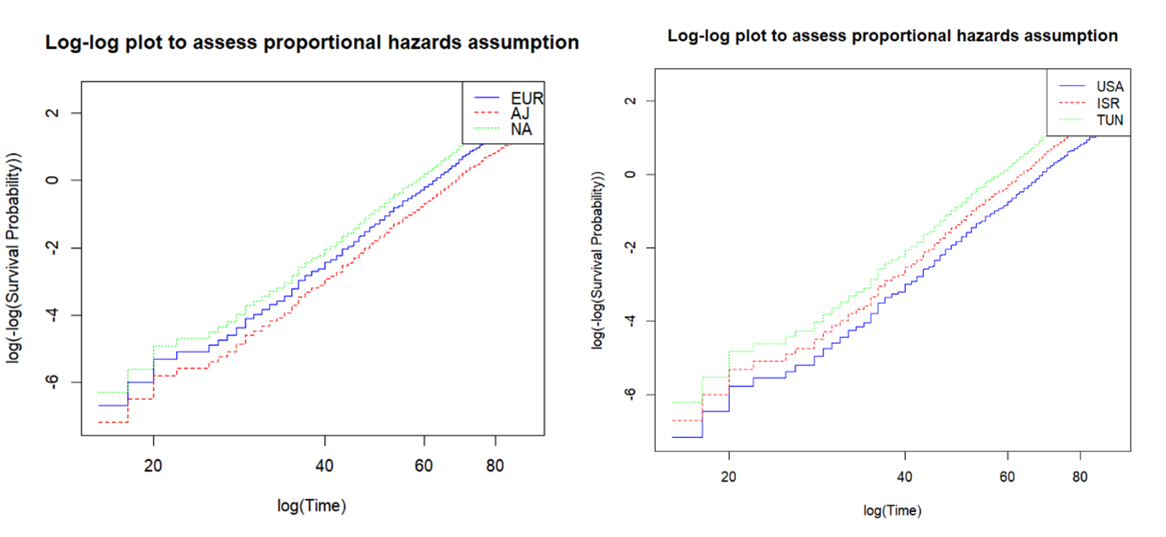


**Supplementary Figure 3. The cumulative incidence of LRRK2 p.Gly2019Ser variant carriers.** The survival curve plot displays the cumulative incidence across the entire cohort, along with the 95% confidence interval for both affected and unaffected *LRRK2* variant carriers.

**Supplementary Figure 2. Log-log plots to test the proportional hazards assumption.** We evaluated the assumption of proportional hazards using log(log(Survival Probability)) versus log(Time) plots and confirmed parallelism of the curves across stratifications by ancestry or country.


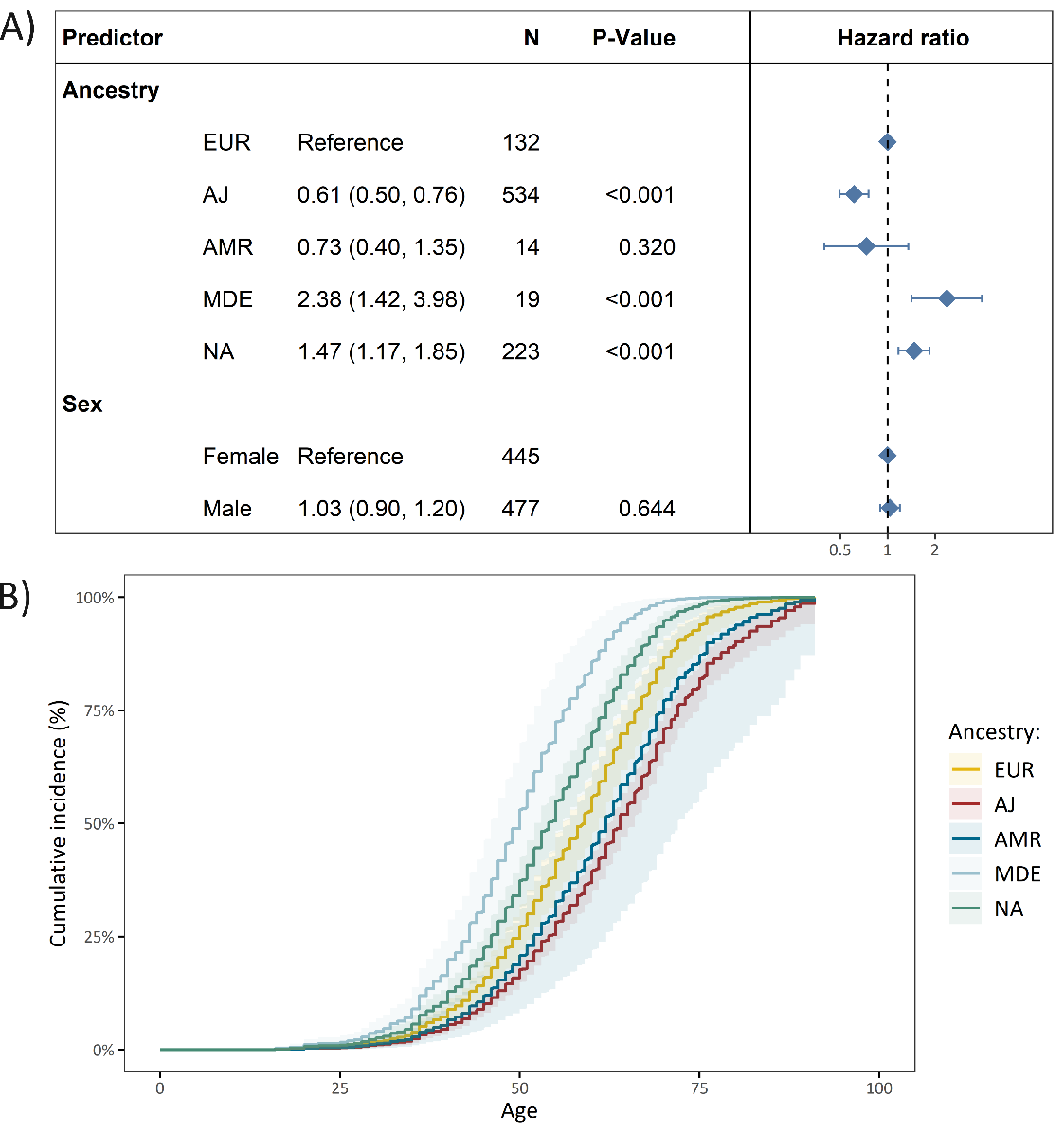


**Supplementary Figure 4. The difference in cumulative incidence of LRRK2 p.Gly2019Ser variant carriers from different genetic ancestries. (A)** The forest plot indicates the difference in cumulative incidence of different ancestries where the hazard ratios, confidence intervals and *P*-values were derived from a Cox proportional-hazards model, adjusted for sex. The reference category of the assessed ancestries was set to European ancestry (EUR). Affected and unaffected LRRK2 p.Gly2019Ser variant carriers were included in the model and the outcome was age at onset or age at examination with right censoring of the affection status. (**B**) Model-based survival curves derived from the sex-adjusted Cox proportional-hazards model are shown. These curves represent sex-adjusted cumulative incidence estimates across ancestries and the 95% confidence interval.

*N*=Number of individuals, Age=Age at examination or age at onset, PD=Parkinson’s disease, AJ=Ashkenazi Jewish ancestry, AMR=Latino and Indigenous Americas populations, MDE=Middle Eastern ancestry, EUR=General European ancestry, NA=North African ancestry.


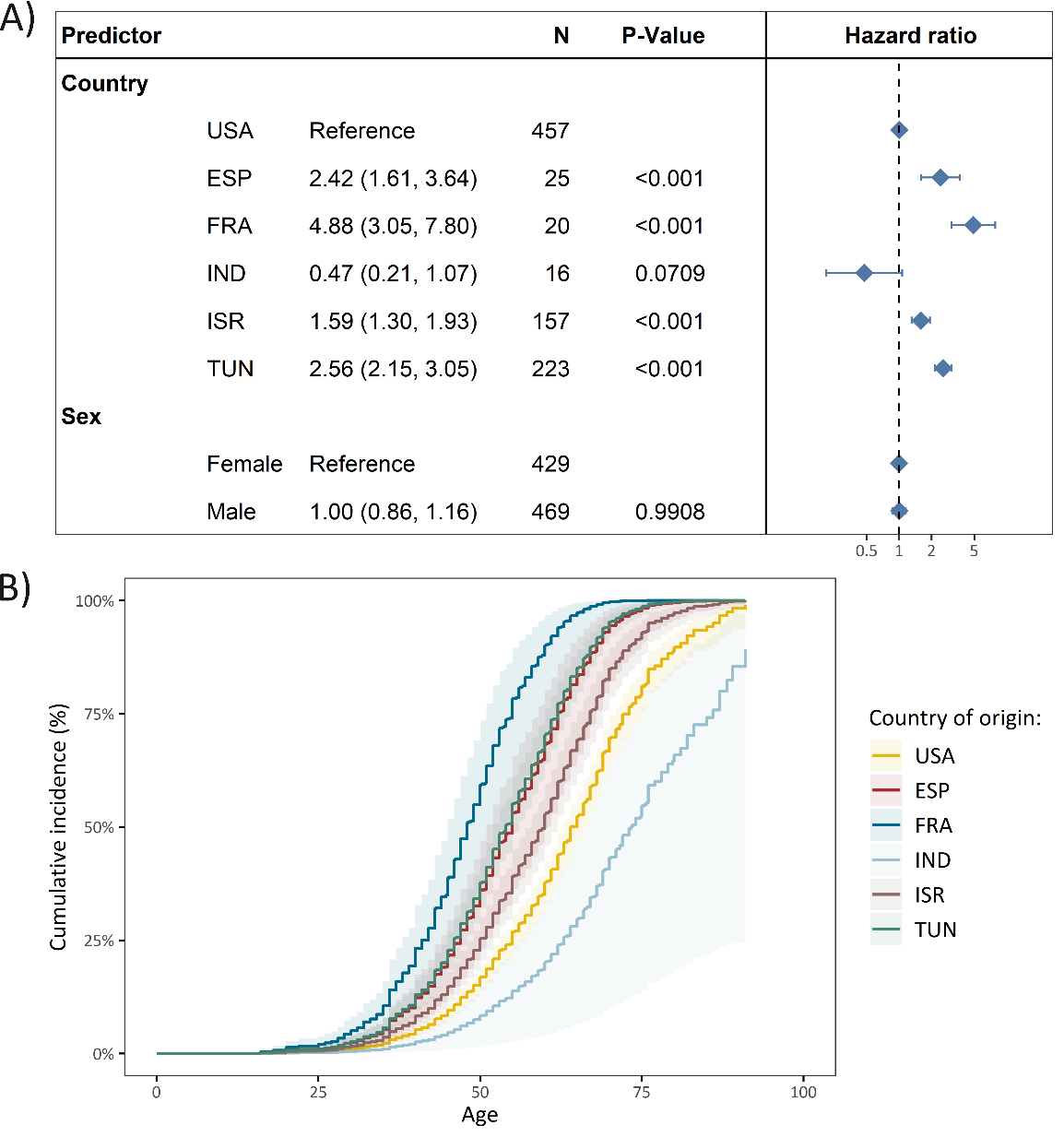


**Supplementary Figure 5. The difference in cumulative incidence of LRRK2 p.Gly2019Ser variant carriers from different countries of origin. (A)** The forest plot indicates the difference in cumulative incidence from different countries where the hazard ratios and *P*-values were derived from a Cox proportional-hazards model, adjusted for sex. The reference category of the assessed countries was set to USA. Affected and unaffected LRRK2 p.Gly2019Ser variant carriers were included in the model and the outcome was age at onset or age at examination with right censoring of the affection status. (**B**) Model-based survival curves derived from the sex-adjusted Cox proportional-hazards model are shown. These curves represent sex-adjusted cumulative incidence estimates across countries and the 95% confidence interval.

*N*=Number of individuals, Age=Age at examination or age at onset, PD=Parkinson’s disease, ESP=Spain, FRA=France, IND=India, ISR=Israel, USA=United States of America, TUN=Tunisia.


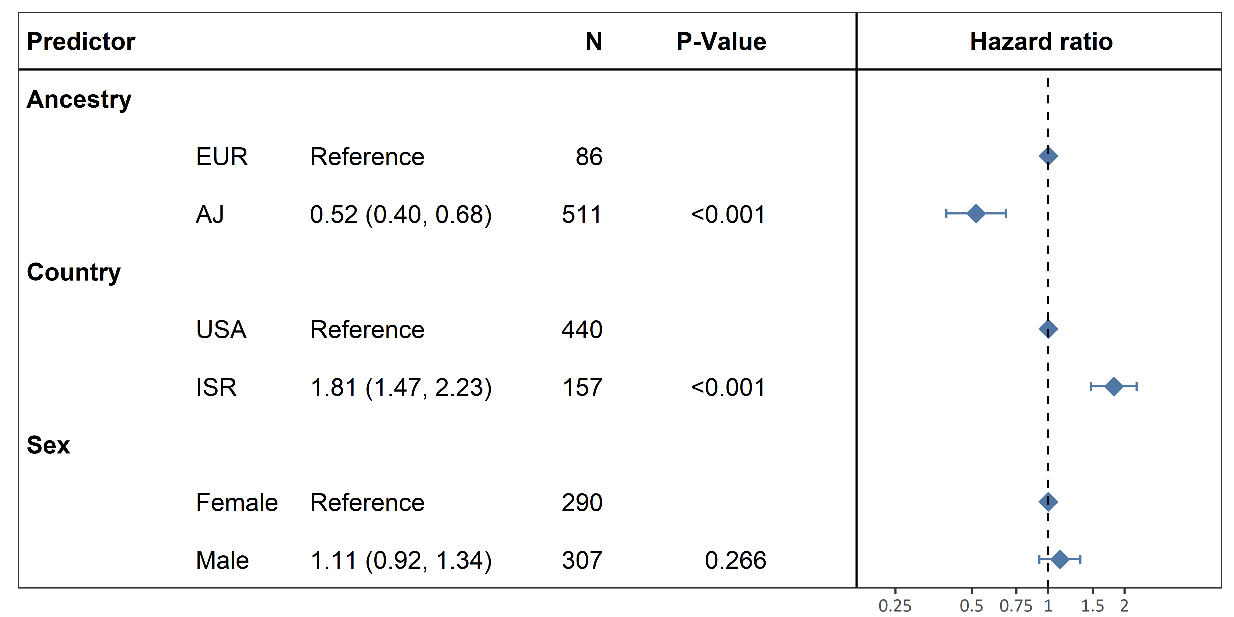


**Supplementary Figure 6. The difference in cumulative incidence of LRRK2 p.Gly2019Ser variant carriers from different genetic ancestries and countries.** The forest plot indicates the difference in cumulative incidence of different ancestries or countries where the hazard ratios, confidence intervals and *P*-values were derived from a Cox proportional-hazards model, adjusted for sex. The reference category of the assessed ancestries or countries was set to European ancestry (EUR) and United States of America (USA), respectively. Affected and unaffected LRRK2 p.Gly2019Ser variant carriers were included in the model and the outcome was age at onset or age at examination with right censoring of the affection status.

*N*=Number of individuals, AJ=Ashkenazi Jewish ancestry, EUR=General European ancestry, ISR=Israel, USA=United States of America.


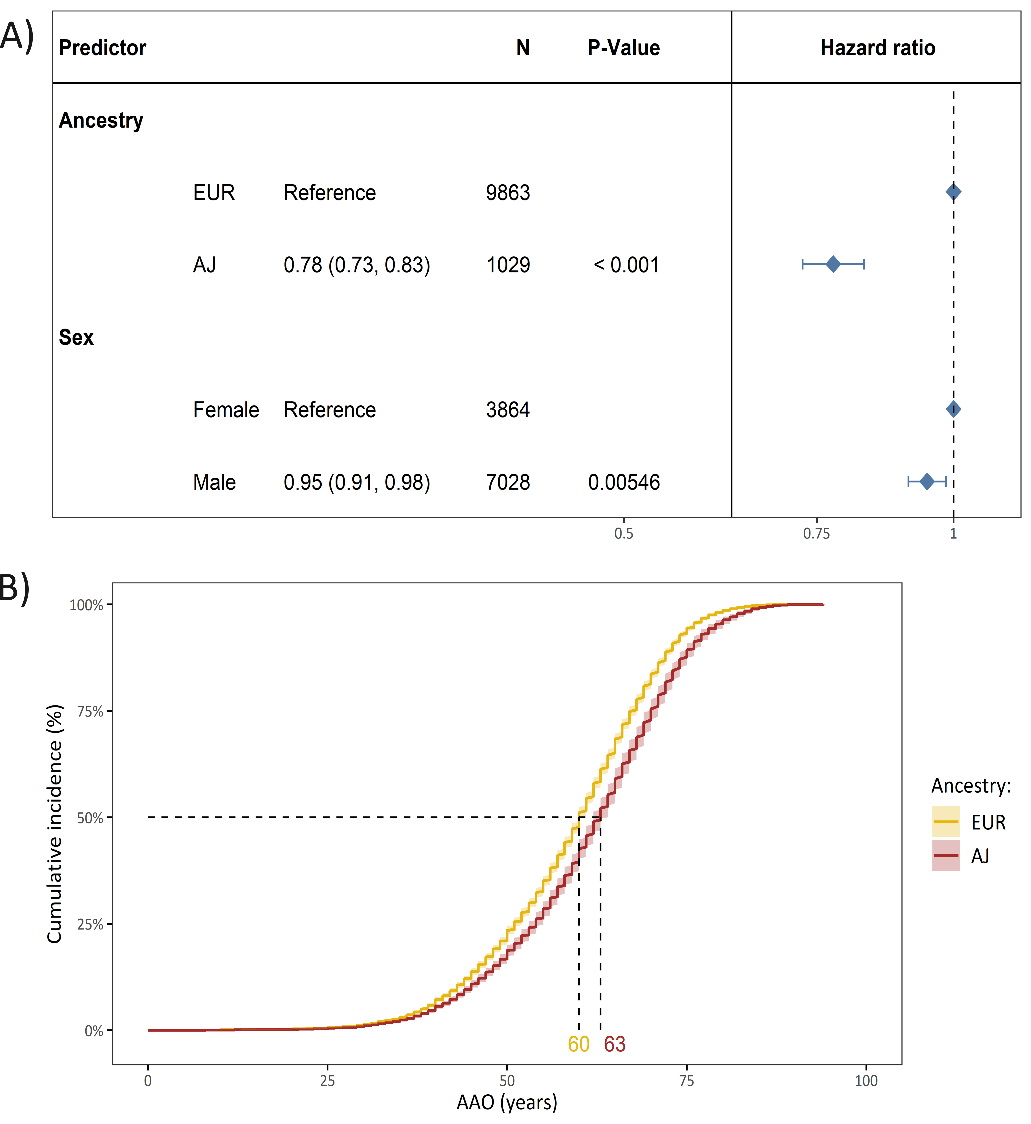


**Supplementary Figure 7. The difference in cumulative incidence of PD patients that do not carry the LRRK2 p.Gly2019Ser from different genetic ancestries. (A)** The forest plot indicates the difference in cumulative incidence of different ancestries where the hazard ratios, confidence intervals and *P*-values were derived from a Cox proportional-hazards model, adjusted for sex. The reference category of the assessed ancestries was set to European ancestry (EUR). (**B**) Model-based survival curves derived from the sex-adjusted Cox proportional-hazards model are shown. These curves represent sex-adjusted cumulative incidence estimates across ancestries and the 95% confidence interval.

*N*=Number of individuals, AAO=age at onset, PD=Parkinson’s disease, AJ=Ashkenazi Jewish ancestry, EUR=General European ancestry.


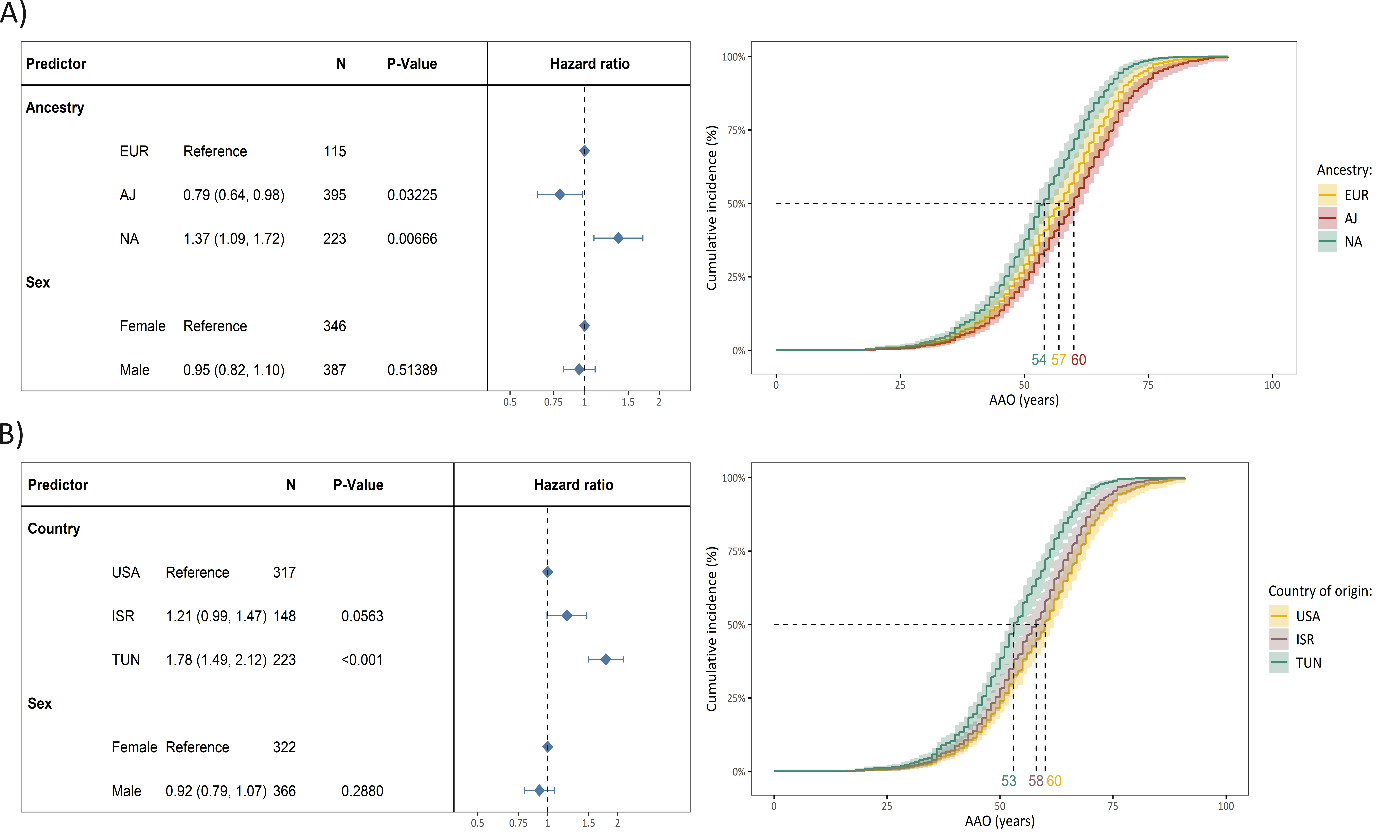


**Supplementary Figure 8. The difference in cumulative incidence of LRRK2 p.Gly2019Ser variant carriers from different genetic ancestries and countries. (A)** The forest plot indicates the difference in cumulative incidence of different ancestries where the hazard ratios, confidence intervals and *P*-values were derived from a Cox proportional-hazards model, adjusted for sex. The reference category of the assessed ancestries was set to European ancestry (EUR). Only affected LRRK2 p.Gly2019Ser variant carriers were included in the model, and the outcome was age at (AAO). Model-based survival curves derived from the sex-adjusted Cox proportional-hazards model are shown. These curves represent sex-adjusted cumulative incidence estimates across ancestries and the 95% confidence interval. **(B)** The forest plot indicates the difference in cumulative incidence of different countries where the hazard ratios, confidence intervals and P-values were derived from a Cox proportional-hazards model, adjusted for sex. The reference category of the assessed countries was set to the United States of America (USA). Only affected LRRK2 p.Gly2019Ser variant carriers were included in the model, and the outcome was age at (AAO). Model-based survival curves derived from the sex-adjusted Cox proportional-hazards model are shown. These curves represent sex-adjusted cumulative incidence estimates across countries and the 95% confidence interval.

*N*=Number of individuals, AJ=Ashkenazi Jewish ancestry, EUR=General European ancestry, ISR=Israel, USA=United States of America, NA=North African ancestry, TUN=Tunisia.


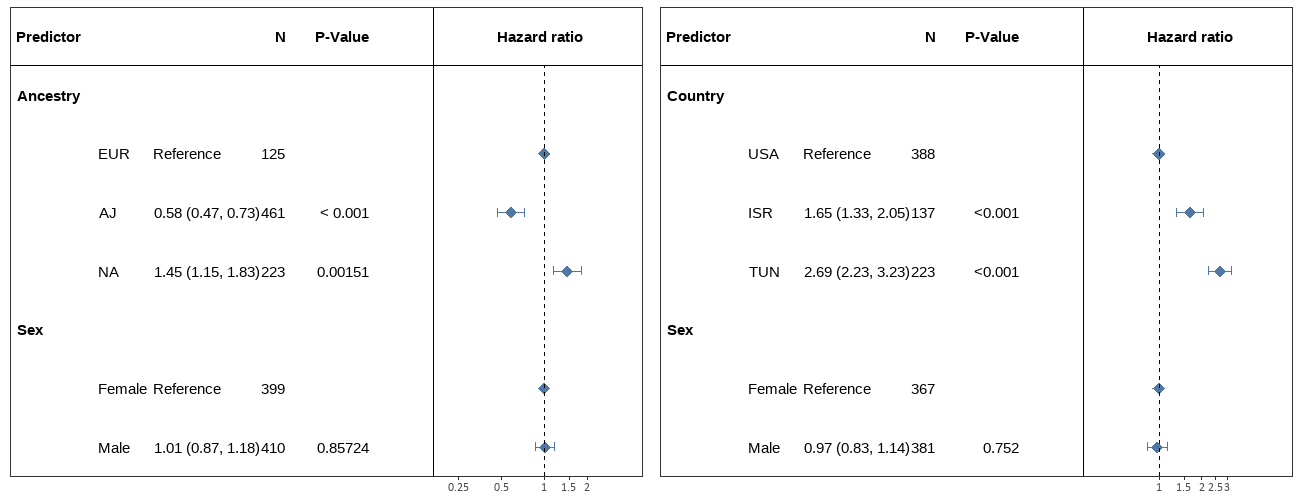


**Supplementary Figure 9. The difference in cumulative incidence of LRRK2 p.Gly2019Ser variant carriers from different genetic ancestry groups and countries of origin.** The forest plots indicate the difference in cumulative incidence from different countries and ancestries, where the hazard ratios and exploratory P-values were derived from a Cox proportional-hazards model, adjusted for sex. The reference category of the assessed countries was set to EUR or the USA, respectively. Affected (only patients with available age at onset) and unaffected LRRK2 p.Gly2019Ser variant carriers were included in the model, and the outcome was age at onset or age at examination with right censoring of the affection status.

N=Number of individuals, Age=Age at examination or age at onset, PD=Parkinson’s disease, ISR=Israel, USA=United States of America, TUN=Tunisia, AJ=Ashkenazi Jewish ancestry, EUR=General European ancestry, NA=North African ancestry.
